# Vitamin D Binding Protein, Total and Free Vitamin D Levels in Different Physiological and Pathophysiological Conditions

**DOI:** 10.3389/fendo.2019.00317

**Published:** 2019-05-28

**Authors:** Daniel David Bikle, Janice Schwartz

**Affiliations:** ^1^Department of Medicine, University of California, San Francisco, San Francisco, CA, United States; ^2^Endocrine Research Unit, San Francisco Veterans Affairs Medical Center, San Francisco, CA, United States

**Keywords:** vitamin D binding protein, vitamin D, free 25(OH)D, free hormone hypothesis, megalin, polymorphisms, liver cirrhosis, pregnancy

## Abstract

This review focuses on the biologic importance of the vitamin D binding protein (DBP) with emphasis on its regulation of total and free vitamin D metabolite levels in various clinical conditions. Nearly all DBP is produced in the liver, where its regulation is influenced by estrogen, glucocorticoids and inflammatory cytokines but not by vitamin D itself. DBP is the most polymorphic protein known, and different DBP alleles can have substantial impact on its biologic functions. The three most common alleles—Gc1f, Gc1s, Gc2—differ in their affinity with the vitamin D metabolites and have been variably associated with a number of clinical conditions. Although DBP has a number of biologic functions independent of vitamin D, its major biologic function is that of regulating circulating free and total levels of vitamin D metabolites. 25 hydroxyvitamin D (25(OH)D) is the best studied form of vitamin D as it provides the best measure of vitamin D status. In a normal non-pregnant individual, approximately 0.03% of 25(OH)D is free; 85% is bound to DBP, 15% is bound to albumin. The free hormone hypothesis postulates that only free 25(OH)D can enter cells. This hypothesis is supported by the observation that mice lacking DBP, and therefore with essentially undetectable 25(OH)D levels, do not show signs of vitamin D deficiency unless put on a vitamin D deficient diet. Similar observations have recently been described in a family with a DBP mutation. This hypothesis also applies to other protein bound lipophilic hormones including glucocorticoids, sex steroids, and thyroid hormone. However, tissues expressing the megalin/cubilin complex, such as the kidney, have the capability of taking up 25(OH)D still bound to DBP, but most tissues rely on the free level. Attempts to calculate the free level using affinity constants generated in a normal individual along with measurement of DBP and total 25(OH)D have not accurately reflected directly measured free levels in a number of clinical conditions. In this review, we examine the impact of different clinical conditions as well as different DBP alleles on the relationship between total and free 25(OH)D, using only data in which the free 25(OH)D level was directly measured. The major conclusion is that a number of clinical conditions alter this relationship, raising the question whether measuring just total 25(OH)D might be misleading regarding the assessment of vitamin D status, and such assessment might be improved by measuring free 25(OH)D instead of or in addition to total 25(OH)D.

## Introduction

Vitamin D enters the body either from its production in the skin or absorption from the intestine. In either case, vitamin D must be transported to tissues such as the liver where it is metabolized to its major circulating form, 25(OH)D, by a variety of enzymes with 25-hydroxylase activity, the major one being CYP2R1. 25(OH)D is then transported to tissues such as the kidney where it gets further metabolized to its biologically active metabolite 1,25 dihydroxyvitamin D (1,25(OH)_2_D) by the mitochondrial based CYP27B1. CYP24A1, found in most tissues, is the major enzyme catabolizing 1,25(OH)2D, thus controlling its impact on a cell specific basis. Vitamin D binding protein (DBP) is the key transport protein which, along with albumin, binds over 99% of the circulating vitamin D metabolites. For most cells it is the unbound 25(OH)D that enters cells (free hormone hypothesis), but at least in some cells such as in the kidney, and likely in the parathyroid gland and placenta, DBP participates in the transport of the 25(OH)D into the cell via a megalin/cubilin complex. Although our focus will be on the transport function of DBP and how that relates to the total and free vitamin D levels in different physiologic and pathophysiologic conditions, DBP has a number of functions independent of its role as a vitamin D transport protein. These functions will be briefly reviewed as they do contribute to the role DBP plays in health and sickness independent of its role in vitamin D transport. DBP is a highly polymorphic protein with at least 120 isoforms distinguished by electrophoresis. Of these, three major isoforms have received the most interest—Gc1f, Gc1s, and Gc2. Their structural differences affect DBP function in ways that have an impact on a number of clinical conditions that will be reviewed.

## Vitamin D Binding Protein

### Genomic Regulation

The human DBP gene is located on chromosome 4q12-q13. It is 35 kb in length and comprised of 13 exons encoding 474 amino acids including a 16 amino acid leader sequence, which is cleaved before release. Numerous tissues express DBP, but the liver is the major source (1). The expression of DBP is increased by estrogen (2) as appreciated with the rise in DBP during pregnancy (3, 4) and with oral contraceptive administration (5). However, the exact mechanism for this induction is not clear as a response element for the estrogen receptor in the DBP promoter has not been identified. Androgens, on the other hand, do not appear to affect DBP expression (2). Dexamethasone and certain cytokines such as IL-6 also increase DBP production, whereas TGFβ is inhibitory (6). As for estrogen, the mechanism underlying such regulation is unclear. However, these cytokines and glucocorticoids are likely to play a role in the increase in DBP production following trauma (after an initial decrease in levels due to actin clearance, see below) (7) and acute liver failure (8), which we will discuss subsequently. Primary hyperparathyroidism, on the other hand, is associated with a reduction in DBP levels, likely contributing to the lower 25(OH)D levels in these patients as the free 25(OH)D is not reduced (9). Vitamin D itself or any of its metabolites do not regulate DBP production (10).

### Structure and Polymorphisms

**Figure 1:**
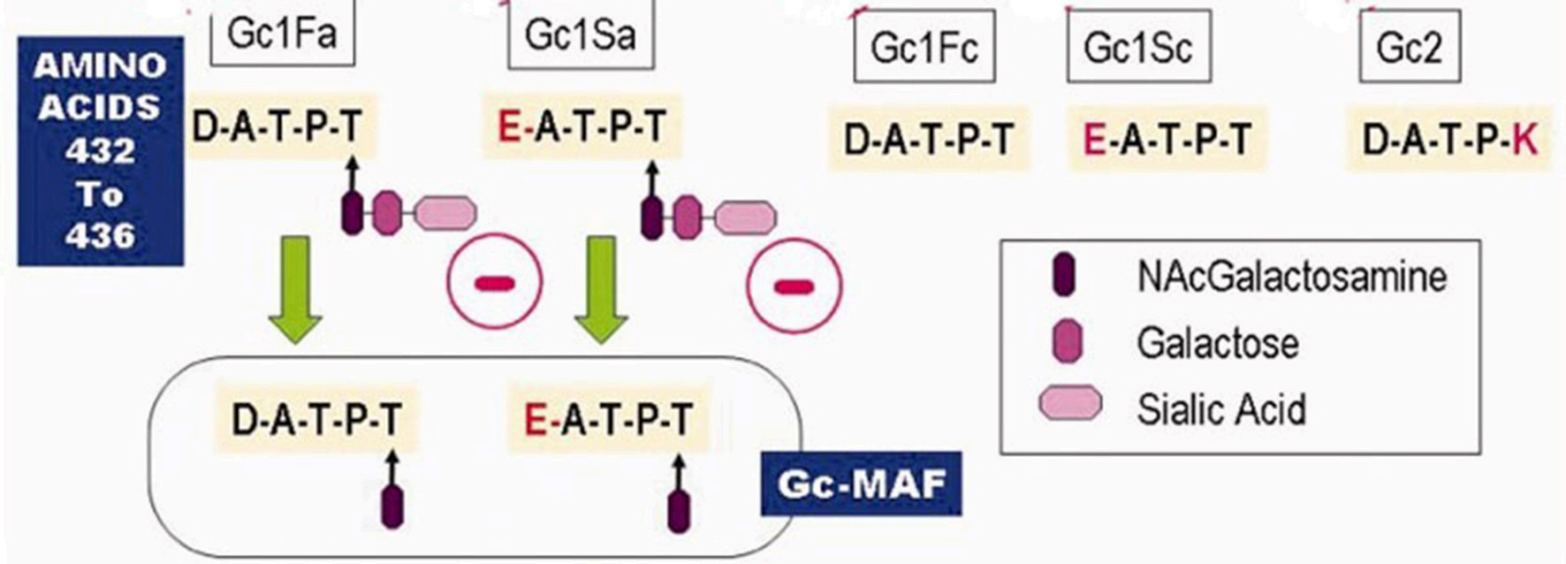
The major DBP alleles. The amino acid differences between the three major DBP alleles are depicted. These differences affect not only their electrophoretic properties but also their glycosylation pattern. In particular Gc2 is not glycosylated, which prevents it from forming the DBP-macrophage activating factor (DBP-MAF). Other biologic differences are discussed in the text.

The mature human DBP is approximately 58 kD in size, although differences in glycosylation of the protein for different alleles alter the actual size. DBP is the most polymorphic gene known. Before the appreciation of its role as a carrier of the vitamin D metabolites these polymorphisms in DBP were used by population geneticists to track different populations, referring to the protein as Gc globulin. Over 120 variants have been described based on electrophoretic properties (11) as noted above with 1,242 polymorphisms currently listed in the NCBI database (12). Of these variants, the Gc1f and Gc1s (rs7041 locus) and Gc2 (rs4588 locus) are the most common (Figure 1). Gc1f and Gc1s involve two polymorphisms, one at aa 432 (416 in the mature DBP) and one at 436 (420 in the mature DBP). The 1f allele encodes the sequence of aa between 432 and 436 as **D**ATPT, the 1s allele encodes the sequence **E**ATPT. This subtle difference in charge makes Gcf run faster (fast) than the Gcs (slow) during electrophoresis. The Gc2 allele encodes DATP**K** which runs slower still. Glycosylation further distinguishes the Gc1 variants from the Gc2 variant. The threonine (T) in Gc1 binds N-acetylgalactosamine to which galactose and sialic acid bind in tandem. The lysine (K) in comparable position in Gc2 is not glycosylated (13, 14). This affects the conversion of DBP to DBP-MAF (macrophage activating factor), which involves a partial deglycosylation removing the galactose and sialic acid by the sequential action of sialidase and β-galactosidase by T and B cells (15). The significance of this for the biologic function is described below.

DBP is comprised of 3 structurally similar domains. The first domain is the binding site for the vitamin D metabolites (aa 35–49). Fatty acid binding utilizes a single high affinity site for both palmitic acid and arachidonic acid, but only arachidonic acid competes with 25(OH)D for binding (16, 17). The actin binding site is located at aa 373–403, spanning parts of domains 2 and 3, but part of domain 1 is also involved (18, 19). The C5a/C5a des Arg binding site is located at aa 130–149 (20). DBP serves as a cochemotactic factor for C51/C5a des Arg in its regulation of neutrophil functions (21). Membrane binding sites have been identified in aa 150–172 and 379–402 (22).

### Biologic Function

#### Binding to and Transport of Vitamin D Metabolites

DBP was discovered by Hirschfeld in 1959 (23), and originally called group specific component (Gc-globulin), but it was not until 1975 that its function as a vitamin D transport protein was appreciated (24). In normal individuals, ~85% of circulating vitamin D metabolites are bound to DBP. Albumin binds ~15% of these metabolites and does so with much lower affinity. Approximately 0.4% of total 1,25(OH)_2_D_3_ and 0.03% of total 25OHD_3_ are free in serum from normal non-pregnant individuals. The affinity of DBP for the vitamin D_2_ metabolites is somewhat less than that for the vitamin D_3_ metabolites (25). The designation of “bioavailable” vitamin D metabolite is the sum of the free vitamin D metabolite and that bound to albumin, thus measuring around 15% in normal individuals [review in (26)]. However, the degree to which the albumin fraction is truly bioavailable is not clear (27). The free hormone hypothesis postulates that only the non-bound fraction (the free fraction) of hormones that otherwise circulate in blood bound to their carrier proteins is able to enter cells and exert their biologic effects. However, at least for some tissues, a transport system has been identified that takes up the 25(OH)D (and presumably other vitamin D metabolites) attached to DBP. That system involves megalin/cubilin.

The role of megalin for vitamin D metabolism was discovered by Nykjaer et al. (28), who found extensive loss of DBP in the megalin knockout mouse and 25(OH)D in its urine. These mice have very poor survival rates. More recently, a kidney specific knockout of megalin was developed with a good survival rate, enabling longer term studies that demonstrated reduced circulating levels of the vitamin D metabolites, hypocalcemia, and osteomalacia (29). Cubilin, together with megalin, forms part of the complex facilitating this transport mechanism [review in (30)]. Other tissues express the megalin/cubilin complex including the parathyroid gland and placenta, but its role outside the kidney has received little interest (30). Moreover, activated monocytes may be able to accumulate DBP by a megalin independent process, although this too needs further study (31, 32).

The physiologic role of DBP is well-illustrated in the DBP knockout mouse. In these mice the vitamin D metabolites are presumably all free and/or bioavailable as albumin levels are normal. Unlike the megalin knockout mice, mice lacking DBP do not show evidence of vitamin D deficiency unless placed on a vitamin D deficient diet despite having very low levels of serum 25(OH)D and 1,25(OH)_2_D and increased loss of these metabolites in the urine (33). Tissue levels of 1,25(OH)_2_D were normal in the DBP knockout mice, and markers of vitamin D function such as expression of intestinal TRPV6, calbindin 9k, PMCA1b, and renal TRPV5 were maintained. Moreover, injection of 1,25(OH)_2_D into these DBP knockouts showed a more rapid increase in the expression of Cyp24A1, TRPV5, and TRPV6 than in DBP intact controls (34). However, on a vitamin D deficient diet they quickly developed vitamin D deficiency. More recently, a family has been described to have a mutation in the DBP gene deleting it from the homozygous patient and decreasing its concentration to 50% of normal in a heterozygous sibling (35). The homozygous patient had nearly undetectable levels of total 25(OH)D, although the free concentration measured directly was comparable to that of the normal sibling, as was that of the heterozygote sibling. Parathyroid hormone, calcium, and phosphate were all normal. Thus, DBP does not appear necessary for getting the vitamin D metabolites into cells, supporting the free hormone hypothesis, but DBP clearly serves as a critical reservoir for the vitamin D metabolites, reducing the risk of vitamin D deficiency when intake or epidermal production is limited.

The DBP alleles have been reported to differ in their affinity to 25(OH)D. Gc1f was initially reported as having the highest affinity and Gc2 the lowest among the common alleles (36), but results from other laboratories have not confirmed these differences, and the results from later studies themselves are inconsistent (37, 38). In one such study evaluating the half life of 25(OH)D in serum, subjects homozygous for the Gc1f allele were found to have the shortest half life indicating a reduced affinity (39). On the other hand, serum containing the Gc1f variant of DBP reduced the ability of 25(OH)D and 1,25(OH)_2_D to induce cathelicidin in monocytes more than that of serum with the Gc2 allele, suggesting the opposite order of affinity (31). Schwartz et al. (40) recently reported that DBP haplotype had significant effects on total 25(OH)D, free 25(OH)D, and DBP levels. The lowest total and free levels of 25(OH)D were seen with the Gc 2/2 haplotype which also tends to have the lowest DBP levels. Other studies have also found lower total 25(OH)D levels in subjects with the Gc2 allele (41–45). The reason the Gc2 allele is associated with lower DBP levels is unknown. DBP haplotype also affected percent free 25(OH)D. The lowest free percentage was seen with the 1s/1s haplotype and the highest one with the 1f/1f haplotype, suggesting that in this survey the Gc1s allele had a higher affinity for 25(OH)D than the Gc1f allele, with the Gc2 allele in between. Furthermore, the different Gc alleles affect the response to vitamin D supplementation. Individuals with the Gc2 variant have been shown to respond to vitamin D supplementation with a more robust increase in 25(OH)D (46). Moreover, within the Gc2 polymorphic region (rs4588), individuals in an Iranian population with an AA genotype within this polymorphic region showed a greater increase in 25(OH)D levels following vitamin D supplementation than those with the GG genotype did (47). Similar results were found with a different polymorphism at rs2282679 in Caucasian women (48). Rs2282679, an intronic polymorphism in the DBP gene that does not alter DBP structure, was previously shown in GWAS studies to be associated with lower 25(OH)D and DBP levels in several different populations (49–51). The clinical significance of these allelic differences is unclear. Differences in these alleles were not found to contribute to a difference in fracture rate in a large study including African Americans and Caucasians (52) or other calcemic and cardiometabolic diseases in the Canadian Multicentre Osteoporosis Study (50). However, as reviewed by Malik et al. (13) and Speeckaert et al. (53), a large number of chronic diseases including type 1 and 2 diabetes (54–56), osteoporosis (57–59), chronic obstructive lung disease (60), endometriosis (61), inflammatory bowel disease (62), some cancers (63–66) [although see (66–68)], and tuberculosis (69) have been associated with DBP variants. Other SNPs at rs4588 have been associated with susceptibility to the metabolic syndrome (70). At the Gc1 locus (rs7041) the G allele is associated with increased susceptibility to hepatitis C viral infection (71). Karras et al. (72) has summarized a number of studies showing the impact of DBP and DBP polymorphisms on various outcomes of pregnancy. These studies demonstrate the recent interest in the impact of polymorphisms on DBP function, but it remains to be seen whether these initial results will be generalized across different populations.

#### Actin Scavenging

A major function of DBP that has received considerably less interest than that of vitamin D metabolite binding is its role in actin scavenging. Following trauma (7), sepsis (73–75), liver trauma (8, 76, 77), acute lung injury (78), preeclampsia (79), surgery (80, 81), and burn injuries (82), large amounts of actin are released from the damaged cells forming polymerized filamentous F-actin that, in combination with coagulation factor Va, can lead to disseminated intravascular coagulation and multiorgan failure unless cleared (83). The actin scavenging system consists of gelsolin and DBP. Gelsolin depolymerizes F-actin to G (globular) actin. DBP, with its high affinity for G-actin (Kd = 10 nM), prevents the repolymerization and clears it from the blood (84, 85). No clear difference among the major DBP variants has been observed regarding binding to G-actin (53). The DBP-actin complexes are rapidly cleared (half life in blood approximately 30 min) (81), primarily by the liver, lungs and spleen. These tissues have receptors for the DBP-actin complexes (86). The acute conditions result in a fall in DBP levels, potentially decreasing the bioavailability of the vitamin D metabolites (8, 87, 88), with a rise in the DBP-actin complexes (7, 73, 77, 78). The ability of the organism to respond to the insult by increasing DBP production is correlated to survival (7, 8, 89), and has led to the consideration of the use of DBP therapeutically (90, 91).

#### Neutrophil Recruitment and Migration With Complement 5a (C5a) Binding

Neutrophil activation during inflammation increases their binding sites for DBP (92), and DBP binding to these sites facilitates C5a induced chemotaxis (21) as well as other chemoattractants such as CXCL1 during inflammation (93).The interaction with C5a involves residues 130–149 of DBP, a region which is common to all major DBP alleles (20), and no difference in these alleles has been found with respect to their promotion of C5a mediated chemotaxis (21). Binding of 1,25(OH)_2_D but not 25(OH)D blocks the promotion by DBP of C5a activity (94).

#### Fatty Acid Binding

DBP binds fatty acids but with lower affinity (Ka = 10^5^-10^6^M^−1^) than albumin and via a single binding site (16, 95). Most of the fatty acids binding to DBP are mono-unsaturated or saturated, with only 5% poly-unsaturated. However, only poly-unsaturated fatty acids such as arachidonic acid and linoleic acid compete with vitamin D metabolites for DBP binding (17, 96). This suggests that the different fatty acids alter the configuration of DBP affecting the binding of the vitamin D metabolites rather than directly competing with the vitamin D metabolites for their binding site. The role of DBP in fatty acid transport appears limited.

#### Formation of the DBP-Macrophage Activating Factor (DBP-MAF) and its Functions

As described above, DBP-MAF is formed from certain alleles (Gc1s and 1f) of DBP following deglycoslyation during inflammatory processes (97). These deglycosylation steps are required for the role of DBP in macrophage activation (15), but further removal of the N-acetyl-galactosamine (NaGal) reduces this activity (98). DBP-MAF is able to activate osteoclasts (99) independent of its 25(OH)D binding function, and it has been shown to stimulate bone resorption in the osteopetrosis (OP) and the incisor absent (IA) rat (100). DBP-MAF has also shown efficacy in a number of tumor models (101–103). Removal of NaGal by α-NaGalase blocks DBP-MAF formation contributing to the loss of immunosuppression in cancer patients (104). α-NaGalase is produced in the liver, and appears to be directly related to tumor burden (105). Preparations of DBP-MAF may have therapeutic potential (14).

## Free Hormone Hypothesis

**Figure 2:**
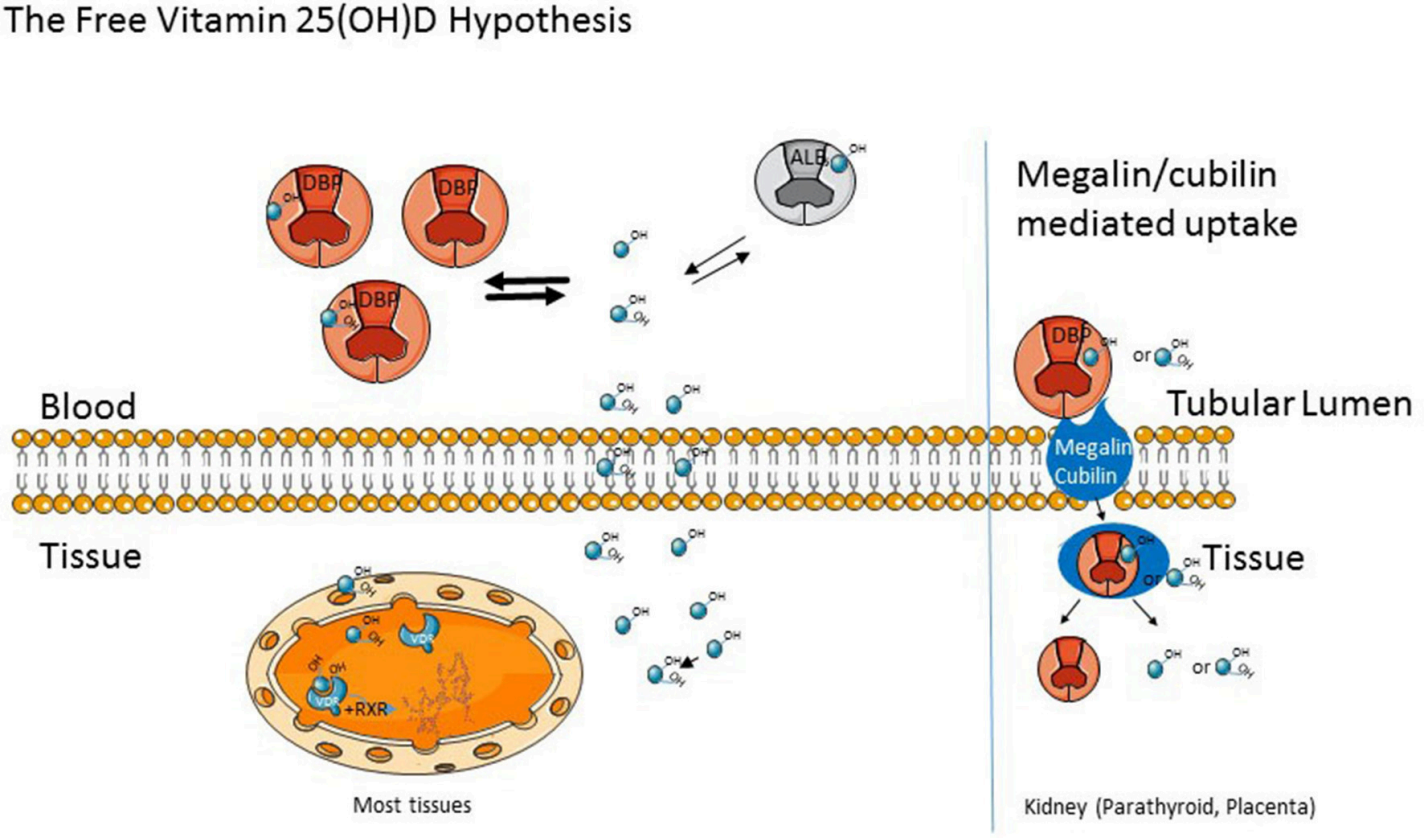
The Free Vitamin D hypothesis. As noted in the text, vitamin D (OH) metabolites are bound to D Binding Protein (DBP) and to a lesser extent albumin in the circulation. These cross the cell membrane as the free (unbound) metabolite in most tissue. However, In the kidney, parathyroid gland, and placenta, the megalin/cubilin complex can transport bound D (OH) metabolites into cells.

As previously noted, the free hormone hypothesis postulates that only the non-bound fraction (the free fraction) of hormones that otherwise circulates in blood bound to their carrier proteins is able to enter cells and exert their biologic effects (Figure 2). Examples include the vitamin D metabolites, which we are discussing in this review, sex steroids, cortisol, and thyroid hormone. These are lipophilic hormones assumed to cross the plasma membrane by diffusion and not by an active transport mechanism. One of the earliest clinical examples leading to the formulation of the free hormone hypothesis came from observations by Recant and Riggs (106) that patients with protein losing nephropathy developed quite low levels of thyroid hormone (PBI) along with increased urinary losses but without evidence of hypothyroidism. Subsequent studies have established the free hormone hypothesis for the thyroid and steroid hormones (107, 108), and measurements of the free concentrations of thyroid hormone, estrogen, and testosterone are standard practice. As will be discussed subsequently, this is likely to become the case for free 25(OH)D. As noted earlier, mice lacking DBP lost substantial amounts of the vitamin D metabolites in the urine with marked reductions in their circulating levels of 25(OH) D, but they did not develop evidence of rickets until put on a low vitamin D diet. Such results indicate the importance of the free fraction of 25(OH)D for biologic functions and the role of DBP as a circulating reservoir (33).

To address the clinical relevance of the free hormone hypothesis for vitamin D metabolites, a method to measure the free concentration needed to be developed. This was originally performed by centrifugal ultrafiltration to directly determine the free levels of 25(OH)D and 1,25(OH)_2_D (109, 110) in various clinical situations. However, this method is labor intensive and has recently been replaced at least for free 25(OH)D by a two-step ELISA that directly measures free 25(OH)D (Future Diagnostics Solutions B.V., Wijchen, Netherlands) using monoclonal antibodies from DIAsource Immunoassays (Louvain-la-Neuve, Belgium). The antibody in the current assay does not recognize 25(OH)D_2_ as well as 25(OH)D_3_ (77% of the 25(OH)D_3_ value), so underestimates the free 25(OH)D_2._ However, under most situations where the predominant vitamin D metabolite is 25(OH)D_3_, the data compare quite well to those obtained from similar populations using the centrifugal ultrafiltration assay (111, 112). The initial studies with the centrifugal ultrafiltration method established affinity constants for DBP and albumin binding to 25(OH)D and 1,25(OH)_2_D in a healthy young adult (DD Bikle) and may not be generalizable to a broad range of individuals from different ethnic backgrounds or in different clinical conditions. However, prior to the development of a high throughput ELISA assay to measure the free concentration directly, these affinity constants proved useful in calculating the free concentrations (113, 114) from measurements of DBP, albumin and the total vitamin D metabolite of interest according to the formula:

$$\begin{array}{l} free\;vitamin\;D\;metabolite=\frac{total\;vitamin\;D\;metabolite}{1+\left(Ka_{alb}*albumin\right)+\left(Ka_{DBP}*DBP\right)} \end{array}$$

As noted previously, the affinity of 25(OH)D for albumin is much less that than for DBP, leading some to consider albumin-bound 25(OHD) to be essentially “free” or “available” and define “bioavailable 25(OH)D” as free 25(OH)D plus albumin-bound 25(OH)D. Given that the albumin bound 25(OH)D (15%) is considerably higher than the free level (0.03%), this would imply that approximately 500 times as much 25(OH)D is available to cells than if only the free fractions were available. There is little evidence to support albumin bound 25(OH)D as being readily available to cells.

In sera from normal healthy younger individuals, the calculated values of free 25(OH)D and 1,25(OH)_2_D using DBP measured with polyclonal antibodies correlate reasonably well with the directly measured free levels using centrifugal ultrafiltration for both metabolites or the ELISA assay for 25(OH)D. However, when applied to clinical populations with altered DBP levels either during physiologic (e.g., pregnancy) or pathologic (eg. liver disease) conditions, the calculated values no longer are consistent with those measured directly by either centrifugal ultrafiltration or the newly developed ELISA (115). Part of this is due to the disparity between assays for both the vitamin D metabolite (e.g., 25(OH)D) and DBP, each of which have generally relied on immunoassays. However, mass spectroscopy is becoming the gold standard for measurement of the vitamin D metabolites (116, 117) and is being developed for the measurement of DBP and its various isoforms as well (42, 118). The adoption of mass spectroscopy should reduce the variation in these measurements from different laboratories. But a major problem in attempting to calculate the free fraction of vitamin D metabolites is the assumption that all DBP alleles have the same affinity for the vitamin D metabolites, and that this is invariant under varying clinical conditions. As noted previously, the rank order of affinity of the different alleles for the vitamin D metabolites remains controversial, but differences have been found. Regardless, these potential differences in measured affinity do not begin to explain the large differences between the calculated and directly measured free metabolite levels in various disease states (40). Although there are statistically significant correlations between calculated and directly measured free 25(OH)D, the relationship accounts for only 13% of the variation. Calculated free 25(OH)D concentrations are consistently higher than directly measured concentrations in a variety of studies, such as those performed during the third trimester of pregnancy and in patients with liver disease or cystic fibrosis (115, 119–122). These studies suggest changes in the affinity of 25(OH)D to DBP independent of allelic variations in at least some of these clinical conditions.

## Clinical Studies

### Healthy Populations

**Figure 3:**
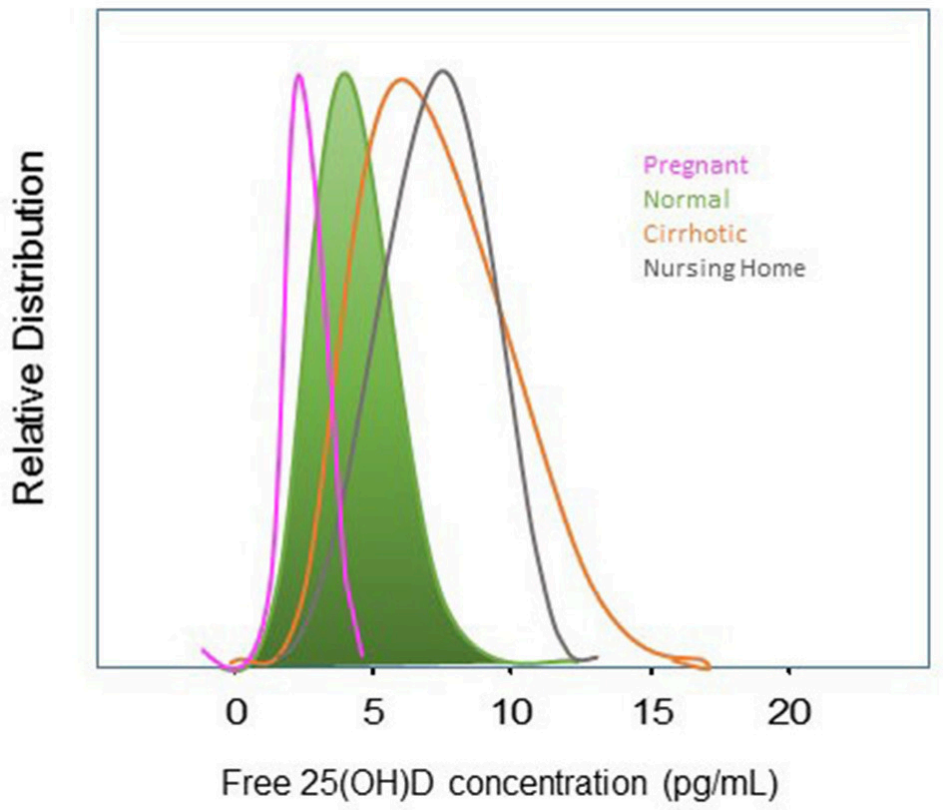
Distribution of free 25(OH)D in Adults and Selected Patient Groups. Distribution of directly-measured free 25(OH)D in normal adults (in green), pregnant women (pink), cirrhotics (orange), and nursing home residents (gray). Distributions are shifted leftward toward lower free 25(OH)D concentrations in pregnant women in the 2nd and 3rd trimesters concordant with increased DBP while decreased synthetic function and DBP in cirrhotics shifts free 25(OH) concentrations to the right toward higher levels. The mechanism for higher free 25(OH) concentrations in Nursing home residents is likely related to D supplementation, somewhat lower, albumin, and the pro-inflammatory state of frailty. Figure generated form data in Schwartz et al. (40).

**Figure 4:**
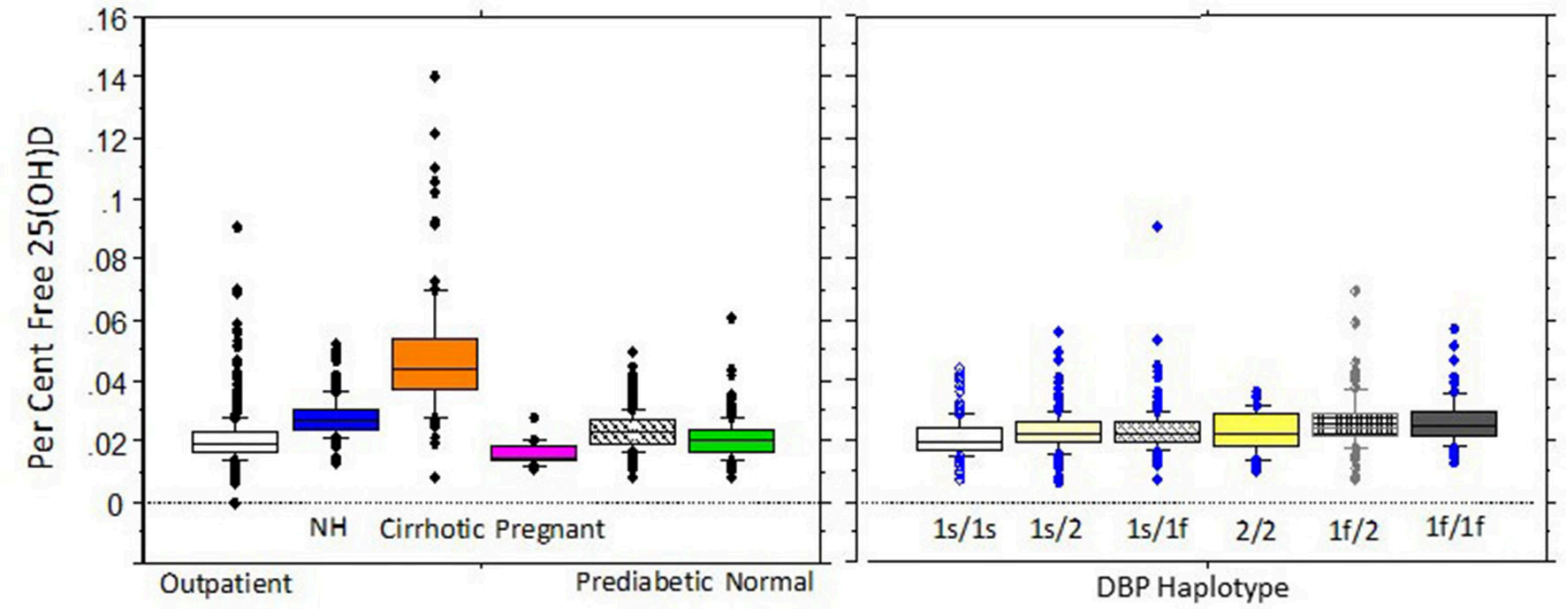
Percent free 25(OH)D in adults by clinical condition or DBP Haplotype. Percent free 25(OH)D concentrations for selected clinical groups on the left panel (community outpatients, NH=nursing home patients, cirrhotics, pregnant women, prediabetics, and normal individuals) and by DBP haplotype on the right . Boxplots show 10th, 25th, median, 75th, and 90th percentile values. Individual points represent values above the 90th and below the 10th percentiles. Both clinical subgroup and DBP genotype significantly effect percentage free 25(OH)D. Between group comparisons for clinical conditions were significant for all but healthy persons compared with pregnant women or outpatients, or for pregnant women compared with outpatients. For DBP haplotypes, smaller but significant differences were detected between the 1s/1s haplotype and the 1s/1f,1f/2, 1f/1f, and 1s/2 haplotypes and between the 1s/2 and 1f/2 and 1f/1f haplotypes and between the 1s/1f and 1f/1f haplotypes. Data are reproduced with permission from Schwartz et al. (40).

Determinations of free 25(OH)D concentrations in healthy populations show highly significant correlations with total 25(OH)D concentrations whether measured directly or indirectly. Assays to directly measure free 25(OH)D are not currently available for use in clinical care but have been used in research investigations. As noted above, calculated 25(OH)D values are usually higher than when measured directly, which is based on multiple unsubstantiated assumptions such that results obtained with the two methods can differ markedly in different clinical conditions. For these reasons only results from studies with directly measured free 25(OH)D will be discussed. When measured with the direct immunoassay, free 25(OH)D levels have been reported to be between 0.02 and 0.09% of total 25(OH)D concentrations and generally range from 0.5 to 8.1 pg/mL in 95% of healthy adults (Figure 3). However, clinical conditions that alter either DBP, the affinity of DBP for 25(OH)D metabolites or albumin, or disposition of vitamin D, may alter free 25(OH)D concentrations or relationships between free and total 25(OH)D concentrations. In this regard, a number of medications, hormones, and smoking have been shown to affect DBP levels (123). Thus, as shown in Figure 3, the free concentration of 25(OH)D varies among different clinical conditions. DBP haplotypes have also been hypothesized to alter the affinity between total 25(OH)D and free 25(OH)D, although, as shown in Figure 4, the variation in percent free 25(OH)D levels is less affected by DBP haplotype than clinical condition.

### Free 25(OH) D in Conditions That Alter DBP

#### Pregnancy

As pregnancy progresses there are time dependent changes in DBP with almost two-fold increases between the second and third trimesters. Despite these marked DBP changes, mean free 25(OH)D may be the same as or only slightly lower than in non-pregnant women but with less variability than in other groups (40, 124). The slope of the free 25(OH)D vs. total 25(OH) D relationship, however, is significantly less steep than in healthy individuals. The same conclusion was drawn from earlier studies with measurements of free 1,25(OH)_2_D (109). These results suggest that the affinity of DBP for vitamin D metabolites is decreased during pregnancy, perhaps compensating for increased DBP concentrations and the needs of both the mother and fetus for calcium.

#### Liver Disease

Liver diseases that are associated with impaired protein synthetic function such as cirrhosis and acute liver failure result in reductions in DBP and albumin. In addition, the relationship between free 25(OH)D and total 25(OH)D is significantly steeper in patients with cirrhosis than in healthy people indicating altered affinity of DBP for 25(OH)D (40) (Figure 4). The net result is that directly measured free 25(OH)D is higher and shows greater variability in patients with cirrhosis compared to healthy individuals and stable outpatients with other chronic conditions (40, 110, 115) despite lower total 25(OH) D concentrations. Results regarding the effects of cirrhosis or acute liver failure on the relationship of total to free 25(OH)D are consistent, creating a strong argument for assessment of free 25(OH)D to assess vitamin D status in the presence of liver pathology as total 25(OH)D measurements may be misleading.

#### Renal Disease

Nephrotic syndrome, acute renal failure, acute tubular necrosis, or chronic kidney disease associated with renal tubular necrosis may have decreased transport capacity for DBP from the glomerular filtrate into the renal tubules. Heavy proteinuria can lead to loss of DBP as well as 25(OH)D in the urine as the maximal transport capacity of the megalin/cubulin system is saturated. Reports in the literature have not included direct measurement of free 25(OH)D in these conditions, but a small study of nephrotics showed lower total and free 1,25(OH)_2_D compared to people with normal renal function (125).

### Clinical Conditions Not Associated With Altered DBP Levels

#### Obesity

High BMIs are associated with reductions in total and free 25(OH)D but not DBP or elimination of half-life measurements of 25(OH)D (126). The underlying mechanism for these changes is unknown but may be related to the pro-inflammatory state and circulating cytokines present in obesity, although increased volume of distribution (into fat) has also been invoked.

#### DBP Haplotypes

Investigations using direct measurements of free 25(OH)D have detected statistically significant but not marked differences in free 25(OH)D concentrations between healthy individuals with the six common DBP haplotypes (Figure 4). This is in contrast to the marked differences between haplotypes reported with calculated free 25(OH)D levels (122, 127). As noted previously with directly measured free 25(OH)D, the lowest free 25(OH)D is seen with the Gc 2/2 haplotype and the highest levels with the 1s alleles. Per cent free was highest with the 1f/1f haplotype in our studies (40) (see Figure 4).

#### Nursing Home Subjects

In a vitamin D dose titration study (128) of nursing home residents, who are older, have more chronic co-morbidities, and receive more medications than younger people or community-dwelling elderly, free 25(OH)D levels rose along with increases in total 25(OH)D. The per cent free was higher than in younger adults. Relationships between free and total 25(OH)D were also steeper than those of normal subjects or younger outpatients suggesting altered affinity of 25(OH)D to DBP in this group. Slightly lower albumin concentrations may have also had a small contribution. Inflammation and/or elevated cytokines that accompany very old age or multiple morbidities may have also contributed to altered affinity of 25(OH)D to DBP in this group (129).

#### Associations With Markers of Vitamin D Biologic Function

PTH is generally found to be negatively correlated with free 25(OH)D as well as total 25(OH)D. Reports variably conclude that one or the other shows a slightly more significant relationship, but neither explains more than a small amount of the variability in the relationship. Moreover, if the megalin/cubilin complex is operative in the parathyroid gland as it is in the kidney, PTH levels may not be able to distinguish between free and total 25(OH)D with respect to biologic action. However, further insight into the impact of free vs. total 25(OH)D on PTH levels may be gained from several recent studies showing that with high dose D supplementation, changes in iPTH were significantly related to changes in directly measured free 25(OH)D but not to changes in total 25(OH)D (128, 130, 131), suggesting that free 25(OH)D might be a better marker of the biologically available fraction at higher total 25(OH)D concentrations or when 25(OH)D is changing. Data on relationships between directly measured free 25(OH)D and bone density or markers of bone turnover are inconsistent.

#### Other Conditions

There are limited data on the effect of oral contraceptives or hormone replacement therapy with estrogen, but free 25(OH)D levels and relationships between total and free 25(OH)D do not appear to be significantly influenced by the use of these agents at currently prescribed dosages and routes of administration. Similarly, stable medical conditions such as hypertension, prediabetes, diabetes, osteoporosis, or mild renal disease do not appear to significantly alter relationships between free and total 25(OH)D.

### Summary of Clinical Studies

The impact of clinical conditions on free 25(OH)D is that the absolute level, the percent free 25(OH)D and the relationship between free and total 25(OH)D concentrations, differ in pregnant women, 336 people with cirrhosis, and elderly people with multiple morbidities compared to normals or community-dwelling outpatients. These relationships are affected to a much smaller extent by BMI in all groups. It is key that while DBP haplotype variation is associated with differences in per cent free 25(OH)D, the DBP haplotype effects are far smaller in magnitude than those of pregnancy, cirrhosis, or very old nursing home residents with multiple chronic conditions. Thus, total 25(OH)D measurements may be misleading in persons with altered total-to-free relationships, although for other clinical conditions the relationship between total and free 25(OH)D may be less affected.

## Contribution to the Field

25(OH)D measurements in the blood currently provide the standard assessment of vitamin D status. Nearly all 25(OH)D circulates as the bound form, with the vitamin D binding protein (DBP) accounting for approximately 85% of the binding, with albumin accounting for most of the rest. However, it is the very small percentage that is not protein bound (0.03% in normal individuals) that is able to cross the membrane of most cells. Conditions that alter levels of DBP or its binding to 25(OH)D alter the relationship between free and total levels. If the free concentration provides a more accurate assessment of vitamin D status, measuring only total 25(OH)D levels may be misleading in situations where the relationship between total and free 25(OH)D levels is altered as in liver disease and pregnancy or in individuals with different DBP alleles. This review examines the impact of different DBP alleles and clinical conditions that do the relationship between free and total 25(OH)D levels, concluding that in a number of clinical situations measuring the free level may provide a better index of vitamin D status than total levels in such situations.

## Author Contributions

All authors listed have made a substantial, direct and intellectual contribution to the work, and approved it for publication.

### Conflict of Interest Statement

The authors declare that the research was conducted in the absence of any commercial or financial relationships that could be construed as a potential conflict of interest.
